# Family and developmental history of female versus male adolescents with ADHD: diagnosis-specific overlap, few gender/sex differences

**DOI:** 10.3389/fpsyt.2023.1072735

**Published:** 2023-07-17

**Authors:** Johanna Waltereit, Jonas Zimmer, Veit Roessner, Robert Waltereit

**Affiliations:** ^1^Department of Child and Adolescent Psychiatry, Medical Faculty Carl Gustav Carus, Technical University Dresden, Dresden, Germany; ^2^Department of Child and Adolescent Psychiatry, University Medical Center Göttingen, Göttingen, Germany; ^3^Department of Child and Adolescent Psychiatry, LWL-Klinikum Marsberg, Marsberg, Germany

**Keywords:** ADHD, gender differences, sex differences, family history, developmental history, clinical diagnostic interview

## Abstract

**Background:**

Gender and sex differences in the development of children and adolescents are commonly found in the psychiatric examination. Family and developmental history is an important part of the clinical diagnostic interview, the basic examination technique. Attention-deficit/hyperactivity disorder (ADHD) is associated with diagnosis-specific markers in family and development history. However, it is unclear to what extent ADHD-specific signs and narratives differ between females and males. The aim of this study was to assess and to compare the family and developmental history profiles of female versus male adolescents with ADHD.

**Methods:**

Data were collected using the clinical diagnostic interview technique from parents of female and male patients diagnosed with ADHD (ICD-10  F90.0, F90.1 and F98.8) between the ages of 12 and 17  years (*n* = 92). The two groups were matched in pairs for sex, IQ and ICD-10 diagnosis (F90.0, F90.1 and F98.8). Interview data were operationalized in three categories: 0 - physiological marker, 1 - subclinical marker, 2 - clinical marker. The two groups were compared with two-way ANOVA.

**Results:**

Information about female in comparison to male adolescents were reported in the parental interview with few differences.

**Conclusion:**

Our study suggests that family and developmental history of the neurodevelopmental disorder ADHD is only poorly influenced by gender or sex.

## Introduction

### Gender and sex differences in diagnosing ADHD

Attention-deficit hyperactivity disorder (ADHD) is a childhood-onset neurodevelopmental disorder, characterized by developmentally inappropriate and impairing inattention, motor hyperactivity and impulsivity, with difficulties often continuing into adulthood (1). Sex is an important genetic and neurobiological modifier of disease and health. Gender is an important behavioral modifier in social situations like the mental status examination.

Gender refers to the socially constructed norms that impose and influence social roles and relationships, whereas sex refers to the neurobiological differences between boys and girls. The development as a girl or a boy in a family influences the children’s way they are parented and people’s reactions to certain behaviors, play styles or interests. Besides underlying genetic and endocrine processes, these social expectations lead to differing behaviors in female adolescents and male adolescents (2). The complex inter-relations between sex and gender occur together in clinical practice.

Gender-related behaviors in adolescents do not influence only parents’ view on children, but also mental health professionals’ (MHP) view on patients with ADHD in daily clinical routine. There is an increasing awareness among experts that females with ADHD show a somewhat different set of behaviors in school, in family life and in clinical settings in comparison to male adolescents with ADHD. These common and typical perceptions in daily clinical routine lead to social expectations on gender-specific behaviors by MHPs. The use of these stereotypes like “the disruptive boy” or “the dreamy girl” among parents and among experts in working with children and adolescents with ADHD takes part in our daily social life (3).

Social gender expectations as well as underlying neurobiological sex-related processes could therefore be linked to the diagnostic process and outcome during the first consultation of an MHP service. Working with females with ADHD highlights special challenges in recognition of this group, possibly caused by female compensatory strategies or developmental differences on the one hand and stereotypical expectations of MHPs, parents or teachers on the other hand (4). A better understanding of the diagnostic view of MHPs in daily clinical routine regarding female in comparison to male patients with ADHD appears important to improve the clinical understanding of early diagnostic recognition and identification of females with ADHD. Nevertheless, many aspects of gender and sex differences in ADHD are still not well understood.

### Diagnostic routines leading to ADHD

Following clinical guidelines, the diagnostic process of potentially psychopathological behavior is typically tripartite including a clinical diagnostic interview, the use of rating scales and the assessment of setting-specific information like school reports (4–6). The clinical diagnostic interview, performed by an MHP with parents (or other persons related to the patient), takes an important part at this early stage in the diagnostic process. This technique based on a recall of memories of certain events, stored in the brains of patients and parents. They are the subjective views of patients and parents and can differ from an objective assessment of described events. During this face-to-face-interview, patients and parents are asked questions on several domains, such as current symptoms, history of present illness, developmental history, personal history, family history, assessed by the professional interviewer (7–10).

In contrast to the clinical diagnostic interview technique, rating scales are a useful and time-saving tool in the process of forming diagnostic hypotheses and treatment monitoring. In everyday clinical practice, however, rating scales should be carefully included in first diagnostic decisions, because rigid adherence to cut-offs can lead to a high number of false-positive and false-negative results. In particular, norms for female patients are often not available or females are in some cases underrepresented (11). When diagnosing ADHD in female adolescents, the clinician’s experience of disorder-specific symptoms as opposed to gender-specific or sex-specific symptoms plays a key role (4).

### Gender and sex differences in prevalence and clinical presentation of ADHD

In non-referred population samples of children and adolescents, ADHD is more common in males than in females with a sex ratio of around 3:1 (12). By contrast, in clinical samples there was found an up to nine times higher prevalence in males (13). These findings suggest that in clinical diagnostic settings male adolescents receive the diagnosis of ADHD even more often than predicted by the higher prevalence in population samples. An explanation for this phenomenon may be attributed to the diagnostic criteria of DSM-V or ICD-10, which may be predominantly suitable for male children and adolescents, but only partially for female children and adolescents or even for adults of both sexes. Are these differences substantial in disorder-specific behaviors and do they influence the clinicians view? This discrepancy highlights on the other hand a potentially large number of unidentified and untreated female adolescents with negative consequences for well-being and psychosocial, educational and clinical outcome (13). These differences may lead to the perception of less symptomatic impairment in female adolescents or a misinterpretation of internalizing behaviors in female adolescents with ADHD (14). On the other hand, stereotypes of the “disruptive boy” and the “dreamy girl” trigger access to treatment in the context of ADHD (15). In addition, MHPs, parents and teachers may experience and respond to the same behavior of female and male children and adolescents in different ways due to gender-related behavioral expectations (16).

### Role of family and developmental history in diagnosing ADHD

ADHD is associated with a specific profile in family and developmental history, but the empirical literature is still scarce in this domain. In a previous work by our group (17), a profile of diagnosis-specific markers in family and developmental history of male adolescents with ADHD compared to neurotypical controls was found. In family history, the parents reported cumulative transgenerational effects of more mental disorders, more psychosocial burden like less years in school and lower school-leaving qualifications and impaired relationships between parents and grandparents in the families with children with ADHD. In developmental history, the parents of children with ADHD reported more conflicts and persistent problems, in particular verbal aggressions toward the child. For pregnancy history and the first year of life of their child with ADHD, mothers reported increased stress and more crying behavior of the child. Male children with ADHD were more likely to struggle with speech and language problems. In primary and secondary school age, parents of male children diagnosed with ADHD reported extensive impairments in social and cognitive functioning, not only in concentration (17).

### Aims of the study

In our previous study, we have investigated family and developmental history of male adolescents with ADHD compared to typically developing male adolescents (17). As we have outlined above, there is a knowledge gap about the picture of ADHD in female adolescents. It is in particular unclear to what extent ADHD-specific signs and narratives in family and developmental history differ between female and male adolescents. Do females with ADHD show a different profile in family and developmental history in comparison to male adolescents? If so, how can the peculiarities of females with ADHD in family and developmental history be characterized?

The aim of this study was to assess and compare family and developmental history profiles of female and male adolescents with ADHD. Here, we investigated 46 pairs of female and male adolescents with ADHD matched for IQ, ICD-10-diagnosis and sex, using a typical set of questions regarding family and developmental history assessed with the clinical diagnostic interview technique.

## Methods

### Parental participants, female, and male adolescents with ADHD

In this study, we investigated family and developmental history of male adolescents and female adolescents with ADHD, by interviewing the parents. In the following, the participants are the parents.

This sample is consisting of two groups of participants (*n* = 92). The first one are parents of a female adolescent diagnosed with ADHD (*n* = 46) and the second one are parents of a male adolescent with ADHD (*n* = 46), matched in pairs for IQ, diagnosis and sex of their children to reduce relevant confounding factors. As female patients with ADHD are rarer than male patients with ADHD in the general population, for each female that could be included in the study population, a male matching partner was searched from the larger pool of potential males with ADHD. If no male matching partner was found, the female was not induced in the study. Twenty-four data sets out of these 46 sets of parents of male adolescents with ADHD used for this study were identical with 24 out of 56 data sets from our previous study comparing male adolescents with ADHD versus neurotypical controls (17). Thus, male adolescents with ADHD were considered as the control group in the current study.

Patients included in the study had at any time between the ages of 12 and 17 the WHO ICD-10 diagnoses F90.0, F90.1 or F98.8, the latter one representing attention deficit without hyperactivity. All diagnoses were made during regular treatment by board-certified specialists at Child and Adolescent Psychiatry, University Hospital Carl Gustav Carus. Twenty-six pairs had the ICD-10 diagnosis F90.0, 14 pairs the diagnosis F98.8 and 6 pairs F90.1. Exclusion criteria in both groups were the presence of comorbid psychiatric or neurological disorders according to ICD-10 chapters F and G or an intelligence quotient (IQ) below 70. Although many patients with ADHD suffer from psychiatric comorbidities, which are important in clinical practice, these were excluded here to provide an unbiased study population. Additional sample characteristics can be found in Table 1.

**Table 1:** Description of the sample.

Characteristics of adolescents with ADHD	ADHD girls (*N* = 46)		ADHD boys (*N* = 46)		Group differences		
	*M* (SEM)	min-max	*M* (SEM)	min-max	*t* ratio	df	*p*
Age at survey (yrs.)	14,44 (3,159)	11–24	15,48 (3,308)	11–21	1.566	89	0.121
Age at diagnostic assessment (yrs.)	10,68 (3,150)	5.5–21	9,076 (3,263)	4–17	2.355	89	0.021
Intelligence quotient (IQ)							
Verbal comprehension index (VCI)	105,1 (11,397)	83–140	108,3 (13,824)	81–128	1.011	63	0.316
Fluid reasoning index (FRI)	103,2 (15,484)	67–129	108,7 (12,797)	75–135	1.504	60	0.138
Working memory index (WMI)	97,97 (13,496)	65–129	96,81 (10,873)	71–117	0.375	63	0.709
Processing speed index (PSI)	100,3 (13,500)	79–129	91,75 (11,311)	68–118	2.726	64	0.008
Full scale IQ	102.2	73–133	101,6 (11,919)	75–132	0.204	90	0.839
	ADHD girls (*N* = 46)		ADHD boys (*N* = 46)		Group differences		
School placement	*n*	%	*n*	%	*t* ratio	df	*p*
Special needs setting	16	34.78%	15	32.61%	0.218	90	0.828
Middle school	24	52.17%	32	69.57%	1.470	88	0.145
High school	11	23.91%	16	34.78%	0.763	88	0.447
Medications for ADHD							
Methylphenidate	24	52.17%	36	78.26%	2.701	90	0.008
Diagnosis of pairs (ICD-10)	*n*	%					
F90.0 (inattentive and hyperactive–impulsive)	26	56.52%					
F98.8 (predominantly inattentive)	14	30.44%					
F90.1 (inattentive, hyperactive–impulsive and conduct disorder)	6	13.04%					
Participants characteristics - the parents	ADHD girls (*N* = 46)		ADHD boys (*N* = 46)		Group differences		
	*n*	%	*n*	%	*t* ratio	df	*p*
Education of mothers							
10 years of education	28	60.87%	26	56.52%	0.426	88	0.671
13 years of education	16	34.78%	17	36.96%	0.214	88	0.829
University degree	15	32.61%	15	32.61%	0.000	88	>0,999
Employment of mothers							
unemployed	0	0.00%	2	4.35%	1.431	88	0.156
part-time employment	22	47.83%	10	21.74%	2.721	88	0.008
full-time-employment	23	50.00%	33	71.74%	2.380	87	0.019
Education of fathers							
10 years of education	24	52.17%	23	50.00%	0.438	86	0.663
13 years of education	15	32.61%	18	39.13%	0.491	86	0.625
University degree	14	30.44%	17	36.96%	0.507	86	0.613
Employment of fathers							
unemployed	4	8.70%	4	8.70%	0.348	88	0.729
part-time employment	2	4.35%	3	6.52%	0.456	88	0.650
full-time-employment	39	84.78%	37	80.44%	0.576	88	0.566

The first group represents female adolescents with a diagnosis of ADHD and the other group male adolescents with a diagnosis of ADHD. Participants of the study were the parents of the adolescents from these two groups. Columns show *M* and detailed results from two-way ANOVA.

*N* (total) = 92. ADHD, attention deficit hyperactivity disorder. Age: in years.

The study has been approved by the Ethics Committee of the University Hospital Carl Gustav Carus (reference number EK 295072016) and was performed in accordance with the ethical standards laid down in the 1964 Declaration of Helsinki and its later amendments. Participants and also their children gave written informed consent to participate.

### Recruitment

#### Recruitment of participants

Parents of adolescents with an ADHD diagnosis were recruited as participants. Adolescents with ADHD were current or former patients at the University Hospital Carl Gustav Carus, Dresden. All participants were contacted by telephone and invited for an interview. Both parents and children provided written informed consent before being included in the study.

#### Interviewers

Medical students from the Medical Faculty Carl Gustav Carus, Technical University Dresden, in their final year of Medical Studies, participated in the project as part of a voluntarily scientific program and without financial benefit. They were thoroughly trained in the “family and developmental history questionnaire” and were always supervised by a board-certified specialist in child and adolescent psychiatry. Interviewers were not blinded for the diagnosis of the patients and were equally distributed among both groups.

#### Interview

Parents and interviewer met for an agreed appointment. Family and developmental history data were collected from at least one parent. Modeling a clinical setting, participant and interviewer were seated face-to-face in an examination room of the hospital. The interviewer asked the questions in the same order as they can be found in Supplementary Table S1 and documented answers of parents word-by-word.

### Measures

#### Family and developmental history questionnaire

The “family and developmental history questionnaire” to study family and developmental history in the clinical examination scenario of the MHP has been described (17) and consists of a selection of relevant and typical questions to be answered in a 45 min interview. These questions are commonly used for diagnostics at several German academic child and adolescent psychiatry departments (Göttingen, Hamm, Leipzig, Mannheim, Tübingen, Würzburg and Dresden) and were aligned with leading textbooks (8–10). Questions and operationalizations of answers are presented in Supplementary Table S1.

#### Operationalization

Operationalization was oriented on the CASCAP-D system, which in turn was developed for children and adolescents on basis of the AMDP System for adult patients in psychiatry and the WHO ICD-10 diagnostic inventory (18). The CASCAP-D system is used to operationalize psychopathological items. These items can be categorized into not present/regular (0), lightly (1), moderately (2) or severely present (3). For the operationalization of patient history data, most items in the “family and developmental history questionnaire” were oriented on the description of deficient or pathological behavior. In this sense, these history items were categorized into physiological marker (0), subclinical marker (1) and clinical marker (2). We decided for this more simple decision tree to ensure a straightforward categorization into three options: (rated as 0) obviously neurotypical behavior, (rated as 2) pathological behavior as described in WHO ICD-10/DSM-V or obviously (from the viewpoint of a board-certified specialist in child and adolescent psychiatry) associated with psychiatric disorder, and (rated as 1) behavior at risk for psychiatric disorder. This more simple categorization is similar to the K-SADS structured interview (19). Some items represented different levels of social function or burden of disease rather than psychopathology. In those cases, representing markers of social or educational functioning or degree of disease burden, history items were —in analogy— categorized into neurotypical psychosocial functioning (0), subclinical psychosocial functioning (1) and clinical psychosocial functioning (2) or no disease burden (0), low disease burden (1) and high disease burden (2), respectively.

#### Collection of metric data

Metric data like birth weight and APGAR scores were obtained from patient files.

#### Collection of IQ data

Intelligence quotient (IQ) was assessed with HAWIK (Hamburg-Wechsler-Intelligenztest für Kinder), the German version of the Wechsler Intelligence Scale for Children (e.g., WISC-IV) (20).

### Statistical analysis

The statistical method to analyze variances, two-way ANOVA, was performed with Prism software (GraphPad, San Diego, CA; United States). Differences were considered statistically significant if *p* < 0.05. As there were already few differences between the two groups, we did not correct for multiple comparisons, in order to prevent a false-negative interpretation of overall absent sex differences. Graphical artwork was created with Prism software. Graphs show mean, standard error of the mean (SEM) and significant results from two-way ANOVA. Tables show mean and significant results from two-way ANOVA. One asterisk in a graph represents *p* < 0.05, two asterisks *p* < 0.01, three asterisks *p* < 0.001. “ns” indicates a non-significant result (*p* > 0.05). Detailed statistical results are deposited in the Supplementary Table S2.

## Results

### Socioeconomic, psychosocial, psychiatric, and medical history of parents and grandparents

**Figure 1:**
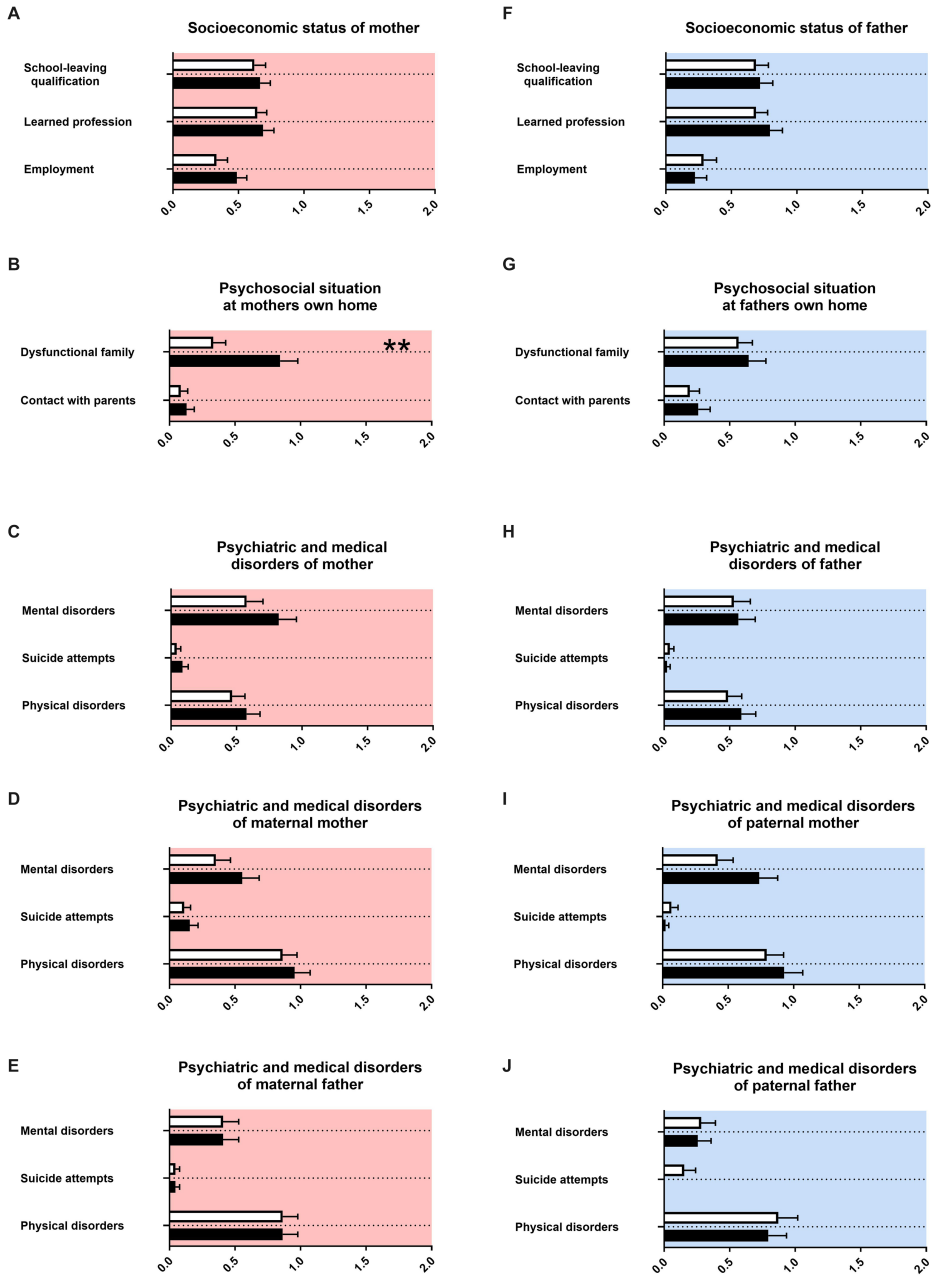
Family history. The x-axis in **(A,F)** represent the level of academic dysfunction (academic, non-academic, no certification) or employment status, respectively (full-time, part-time, no employment). The *x*-axis in **(B–E)** and **(G–K)** represent the level of psychiatric, psychosocial or medical burden. Graphs show mean, SEM and results from two-way ANOVA. Black symbols represent female adolescents with ADHD and white symbols male adolescents with ADHD. Red color in the plotting area represents data about mothers of patients and blue color data about fathers of patients. Two asterisks represents *p* < 0.01.

In the first part of the interview, regarding family history, we collected information using parental memories about themselves and about the grandparents of the child. We assessed socioeconomic, psychosocial, psychiatric and medical history of parents and grandparents. For almost all items, parents of female adolescents and male adolescents remembered similar information about themselves and grandparents, irrespective of the sex of their child (Figure 1). As the only exception, mothers of female adolescents with ADHD were more often described as having a dysfunctional family compared to mothers of male adolescents with ADHD (Figure 1B). The obstetrical history of mothers with female adolescents and male adolescents with ADHD showed no differences (Table 2A). Detailed information about statistical analyses can be found in Supplementary Table S2.

**Table 2:** Developmental history – metric data.

		ADHD girls	ADHD boys				
		*M* (SD)	*M* (SD)	Significance	*p* Value	*t* ratio	df
A	Obstetrical history of the mother						
	Number of pregnancies (*n*)	2,98 (1,56)	2,58 (1,29)	ns	0.193	1.312	88
	Number of childbirths (*n*)	2,44 (0,80)	2,18 (1,17)	ns	0.214	1.253	88
B	Caregivers and family members living with the child at age 12–17						
	Number of biological parents (*n*)	1,48 (0,61)	1,61 (0,54)	ns	0.285	1.076	90
	Number of social parents (*n*)	0,24 (0,45)	0,12 (0,43)	ns	0.638	0.471	90
	Number of grandparents (*n*)	0,00 (0,00)	0,02 (01,4)	ns	0.320	1.000	90
	Number of other adult caregivers (*n*)	0,11 (0,32)	0,07 (0,73)	ns	0.715	0.366	90
	Number of biological siblings (*n*)	0,72 (0,63)	0,76 (0,90)	ns	0.792	0.265	90
	Number of half siblings (*n*)	0,26 (0,48)	0,11 (0,64)	ns	0.204	1.278	90
	Number of social siblings (*n*)	0,04 (0,29)	0,00 (0)	ns	0.320	1.000	90
	Number of peers in youth welfare institutions (*n*)	0,20 (1,31)	0,41 (1,94)	ns	0.535	0.623	90
	Complete number of all persons living together with the child at the age 12–17 (*n*)	3,04 (1,84)	3,17 (2,02)	ns	0.750	0.320	90
	Only child family (*n*)	0,28 (0,40)	0,20 (0,45)	ns	0.334	0.972	90
	Separated parents	0,50 (0,47)	0,33 (0,50)	ns	0.092	1.702	90
	Youth welfare institution	2,17 (0,25)	6,52 (0,15)	ns	0.312	1.017	90
C	Birth parameters						
	Age of mother at childbirth (years)	28,35 (5,19) yrs.	28,74 (4,85) yrs.	ns	0.712	0.370	90
	Age of father at childbirth (years)	33,56 (6,12) yrs.	32,69 (5,48) yrs.	ns	0.486	0.700	88
	Gestational age (weeks)	39,15 (2,40) wks.	38,84 (3,03) wks.	ns	0.593	0.536	88
	Birth weight (*g*)	3,237,05 (773,04) g	3,332,44 (706,68) g	ns	0.549	0.601	87
	Birth size (cm)	49,50 (3,25) cm	50,45 (3,35)cm	ns	0.183	1.342	86
	APGAR 5 min (score)	9,35 (0,67)	9,17 (0,82)	*	0.039	2.094	79
	APGAR 10 min (score)	9,90 (0,37)	9,54 (0,80)	**	0.012	2.578	79
D	Markers of development (0–6 years)						
	Breastfeeding (months)	7,16 (9,10) mo.	6,09 (5,01) mo.	ns	0.496	0.683	87
	First free steps (months)	13,11 (2,71) mo.	13,31 (4,01) mo.	ns	0.780	0.280	89
	First words (months)	12,86 (4,43) mo.	13,70 (4,66) mo.	ns	0.401	0.844	83
	Right-handedness (%)	78%	87%	ns	0.276	1.095	90
	Ambidexterity (%)	13%	9%	ns	0.508	0.664	90
	Left-handedness (%)	9%	4%	ns	0.404	0.839	90
E	School performance in primary school (6–10 years)						
	Repetition of one class (%)	28.26%	34.78%	ns	0.506	0.668	90
	School change (%)	28.26%	28.26%	ns	>0,999	0.000	90
	Regular primary school (%)	74.00%	71.74%	ns	0.817	0.232	90
	Special education settings (%)	34.80%	32.60%	ns	0.828	0.218	90
	Special education in a special school (%)	23.91%	21.74%	ns	0.806	0.246	90
F	Transition into secondary school (10–17 years)						
	High school (%)	27.27%	34.78%	ns	0.447	0.763	88
	Middle school, high performance (%)	54.55%	69.57%	ns	0.145	1.470	88
	Alternative or private school (%)	9.09%	6.52%	ns	0.654	0.450	88
	Special education in secondary school (%)	13.64%	8.70%	ns	0.462	0.740	88
	School change (%)	22.73%	30.43%	ns	0.414	0.820	88

Metric data assessed with the “family and developmental history questionnaire” represent milestones of development and other social and biological markers.

The first group represents female adolescents with a diagnosis of ADHD and the other group male adolescents with a diagnosis of ADHD. Participants of the study were the parents of the adolescents from these two groups. Columns show *M* and detailed results from two-way ANOVA. One asterisk represents *p* < 0.05, two asterisks *p* < 0.01 and three asterisks *p* < 0.001.

### Family relationships, conflicts in the family, maternal stress, and medical history of the child

**Figure 2:**
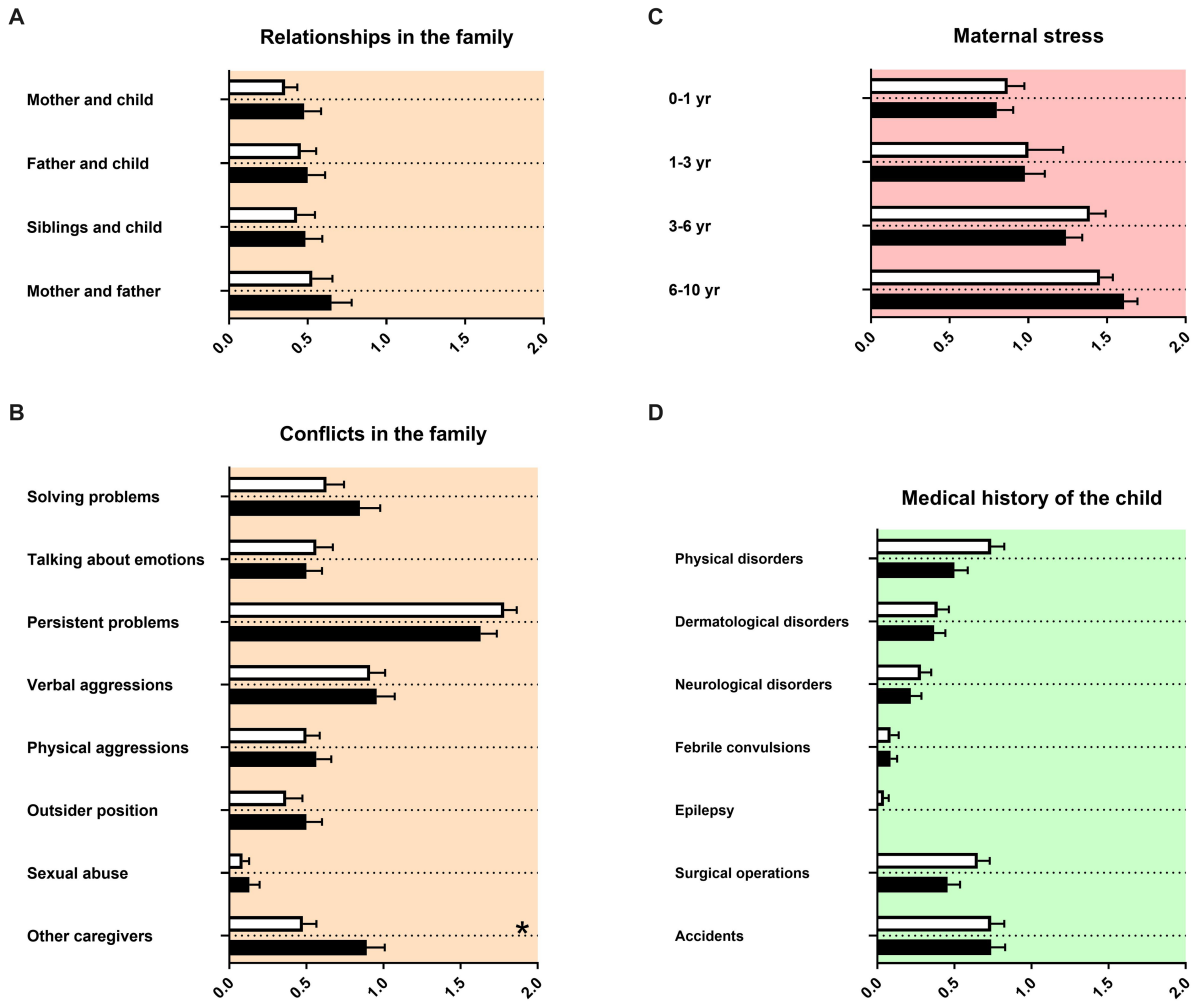
Family relationships and conflicts and medical history of the child. The *x*-axis in **(A–C)** represent the level of psychosocial burden. The *x*-axis in **(D)** represents the level of medical burden. Graphs show mean, SEM and results from two-way ANOVA. Black symbols represent female adolescents with ADHD and white symbols male adolescents with ADHD. Red color in the plotting area represents data about mothers of patients, orange color data about the whole family of patients and green color data about the patients themselves. One asterisk represents *p* < 0.05 and two asterisks *p* < 0.01.

We then assessed family relationships, conflicts in the family, maternal stress and medical history of the child. Parents of female adolescents and male adolescents with ADHD had for most items similar memories, irrespective of the sex of their child (Figure 2). For female adolescents with ADHD in contrast to male adolescents with ADHD, there was more support reported by caregivers who does not live in the household of the family (Figure 2B). The composition of persons living in the family household during late childhood was equal for female adolescents and male adolescents with ADHD (Table 2B).

### Prenatal and perinatal history, birth parameters, and early childhood

**Figure 3:**
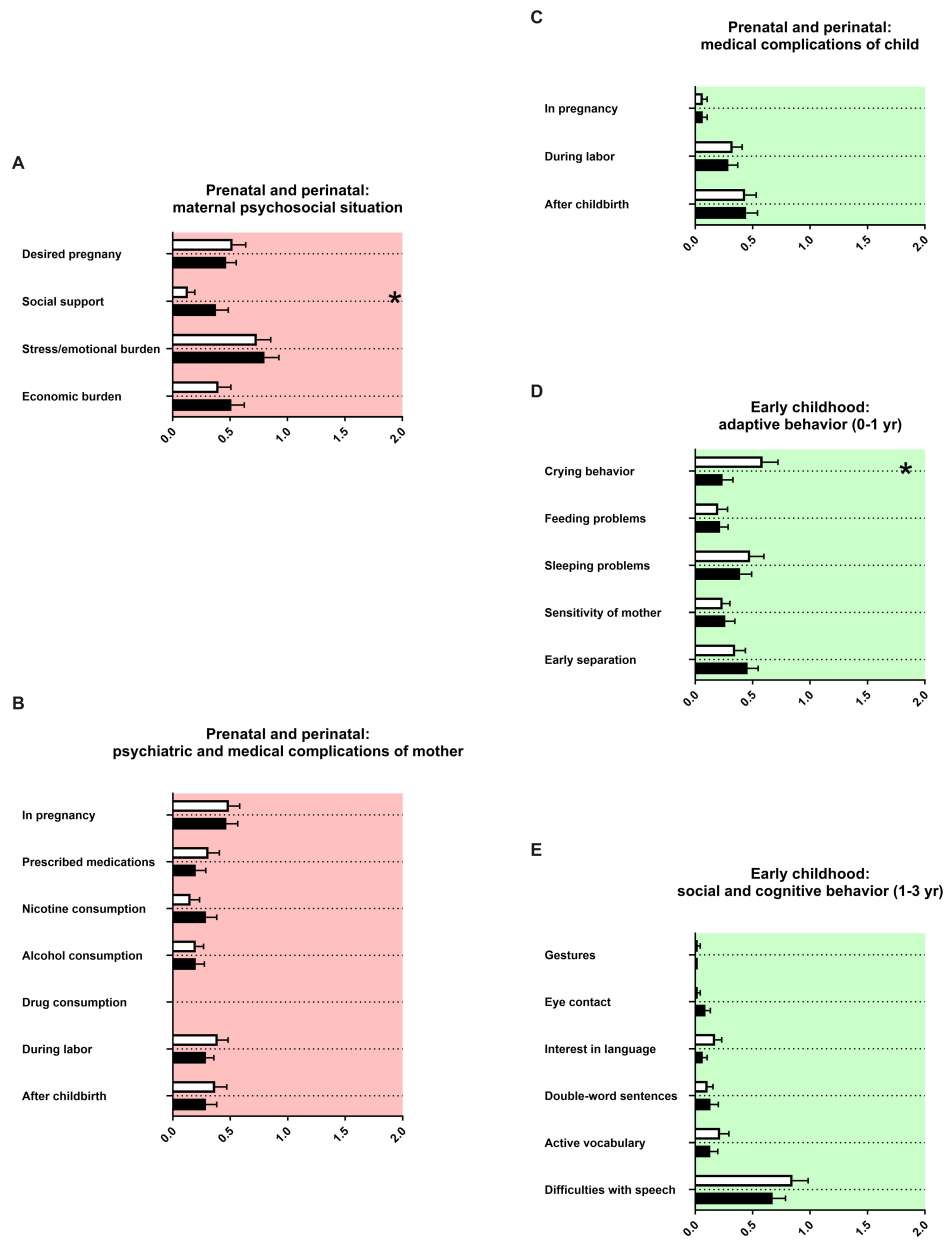
Prenatal and perinatal history and early childhood. The *x*-axis in **(A)** represents the level of psychosocial burden. The x-axis in **(B)** represents the level of psychiatric and medical burden. The *x*-axis in **(C)** represents the level of medical burden. The *x*-axis in **(D)** represents the level of maladaptation between mother and child. The x-axis in **(E)** represents the level of social and cognitive dysfunction. Using gestures and interest in language (songs, rhymes or books) were remembered for the first birthday of the child, eye contact for the first year of life. Double-word-sentences and active vocabulary were remembered for the second birthday. Difficulties with speech represent the use of speech therapy. Graphs show mean, SEM and results from two-way ANOVA. Black symbols represent female adolescents with ADHD and white symbols male adolescents with ADHD. Red color in the plotting area represents data about mothers of patients and green color data about the patients themselves. One asterisk represents *p* < 0.05.

For prenatal and perinatal history, birth parameters and early childhood we observed again strong similarities in the memory of parents of both female adolescents and male adolescents (Figure 3). There were two effects of sex. For mothers of female adolescents with ADHD, less social support was reported during pregnancy (Figure 3A). For male adolescents with ADHD, more crying behavior was remembered during the first year of life (Figure 3D). The APGAR score was decreased for male adolescents with ADHD in comparison to female adolescents with ADHD (Table 2C). Otherwise, birth parameters and markers of development were not reported differently between female adolescents and male adolescents (Tables 2C,D).

### Middle childhood history and late childhood history

**Figure 4:**
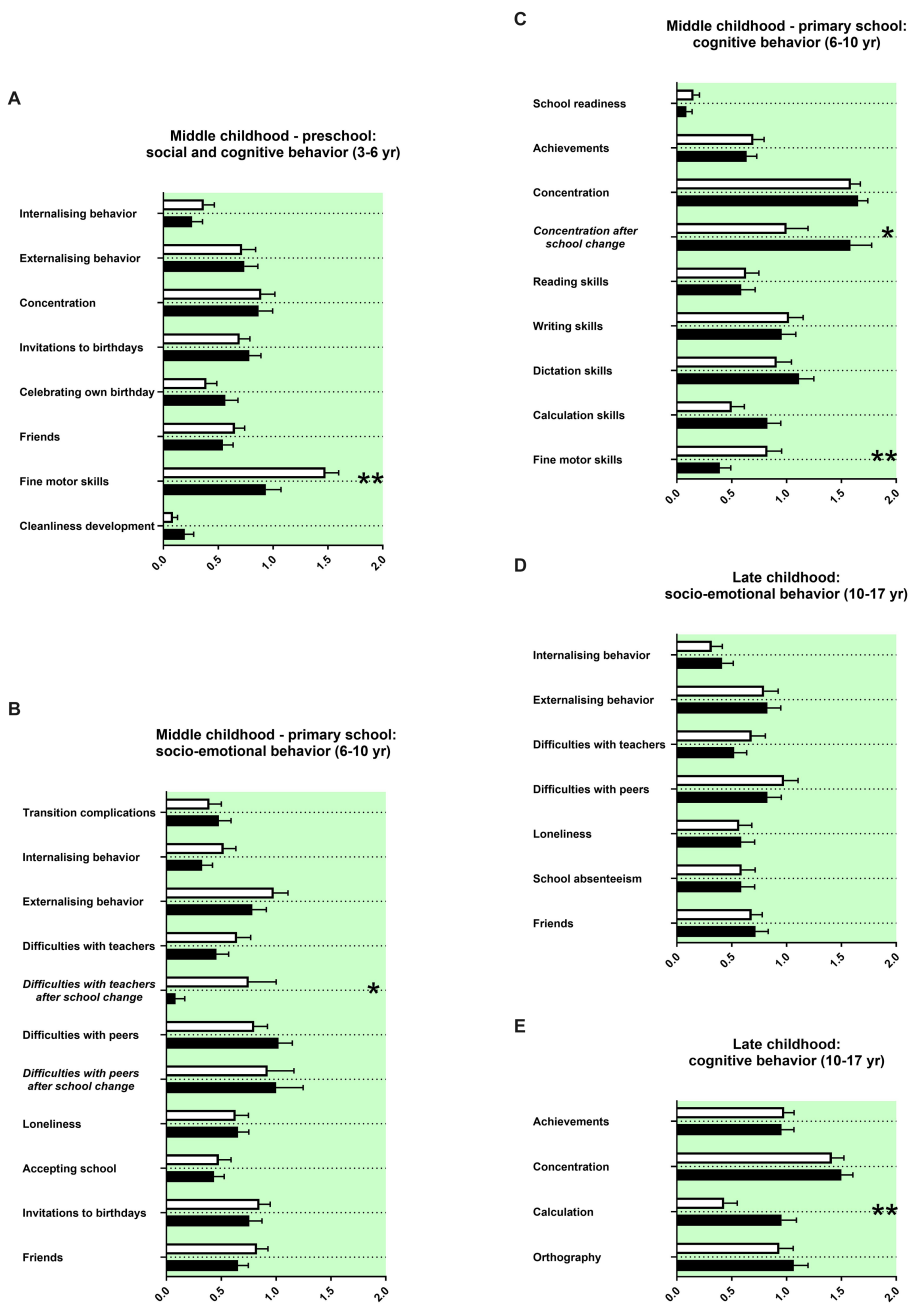
Middle and late childhood history. The *x*-axis represents the level of socio-emotional or cognitive burden **(A–E)**. Graphs show mean, SEM and results from two-way ANOVA. Black symbols represent female adolescents with ADHD and white symbols male adolescents with ADHD. Green colour in the plotting represent data about the patients themselves. One asterisk represents *p* <0.05 and two asterisks *p* <0.01.

Finally, we assessed the socio-emotional and cognitive behaviors of female and male patients with ADHD during preschool, primary school and secondary school. Again, most items were not remembered with a sex difference (Figure 4; Tables 2E,F). Mothers of male adolescents with ADHD reported lower fine motor skills than mothers of female adolescents with ADHD and were more likely referred to occupational therapists during preschool age and primary school age (Figures 4A,C). The parents reported male adolescents with ADHD to show more difficulties with teachers after a school change in primary school (Figure 4B). The concentration in primary school was, however, in female adolescents with ADHD more impaired than in male adolescents with ADHD after a school change (Figure 4C). For secondary school, parents of female adolescents with ADHD reported poorer calculation skills (Figure 4E).

## Discussion

We investigated here family and developmental history in matched pairs of female adolescents with ADHD compared to male adolescents with ADHD. Remarkably, we found here a large overlap in family and developmental history among female and male adolescents with ADHD. There were only few differences, most of them appearing not to be related to the disorder, but to the development as a boy or a girl.

### Overlap between gender/sex in family and developmental history

Family history is important for the diagnostic process of ADHD as it reveals information about the burden of mental disorders and other diseases in the family, conflicts and relationships in the family and socioeconomic background. Developmental history reveals information about the development of psychopathological markers and finally about symptoms of ADHD. In our previous study of family and developmental history, male adolescents with ADHD were compared to neurotypical adolescents without psychiatric diagnosis (17). In this previous study we found for male adolescents with ADHD a diagnosis-specific profile relating to family and developmental history.

The main finding of the present study is that family and developmental history in ADHD is only poorly influenced by gender or sex. In turn, the picture of female adolescents with ADHD is very typical for the disorder. This is on first glance surprising, because the current scientific view emphasizes the importance of gender/sex issues, in particular here in mental healthcare of children and adolescents (2–4). An explanation could be that neurobiological mechanisms in the neurodevelopmental disorder ADHD are relatively stable from gender/sex influences. This may be relevant in particular for the genetic and environmental background of an individual, represented by family history, as well as brain development, represented by developmental history.

### Gender/sex differences in our sample

There were still a couple of gender/sex differences in our findings. As we did not correct here for multiple comparisons – in order not to mask remaining differences, besides the main finding of great overlap in the data set – most of these few differences would disappear when applying alpha correction.

For mothers of females with ADHD, more dysfunctional interaction in the mother’s family was reported (Figure 1B). This has not been described in the literature so far. It may be speculated that dysfunctional family relationships could be better remembered or recognized in the context of females’ families. Gender/sex effects could be recognized in our sample for females as having less adult caregivers than males (Figure 2B). For female adolescents with ADHD it is described to form damaging peer relationships, for example joining an antisocial peer group or engaging in risky sexual practices and partners instead of forming potentially protective relationships to caregivers like teachers.

Lower Apgar scores were reported in our sample for males with ADHD (Table 2C). Between Apgar scores and the diagnosis of ADHD no correlation was described in the literature (21). By contrast, lower Apgar scores were described for male full-term neonates (22). For mothers of females with ADHD, less social support during pregnancy was remembered compared to mothers of males with ADHD (Figure 3A). Less social support is part of long-term stressful life events during pregnancy. The literature on this specific issue is scarce. Prenatal stress of the mother seems however to be more harmful for males than for females (23). Mothers of females with ADHD in our sample reported less frequent and less prolonged crying behavior during the first year of their child’s life than mothers of males with ADHD (Figure 3D). ADHD symptoms in children younger than 3 years have only been sparsely investigated, with main findings of delayed early motor development (24). Increased irritability and increased crying behavior in males or mixed samples with ADHD were reported by mothers in the first year of life of their babies (25–27). Gender and sex differences are, to our knowledge, not investigated.

For middle childhood, parents reported more impaired fine motor skills for males with ADHD during preschool and primary school ages (Figures 4A,C). Female gender/sex is in typically developing children associated with earlier accomplishment of fine motor items (28). Thus, the finding appears more likely associated with gender/sex and not with the neurobiological mechanisms of ADHD. In comparison to males with ADHD, females with ADHD were reported to have substantially less difficulties with teachers after a school change (Figure 4B). In contrast, difficulties with concentration of females with ADHD stayed at a consistently high level after changing school (Figure 4C). However, these data must be interpreted with caution because the number of patients changing school was low (for both females and males 28,3%, see Table 2E). Nevertheless, interesting tendencies can be seen: while these findings may fit into the social stereotypes of the “externalizing and disruptive boy” and “the dreamy girl who does not take medications for ADHD” (4). Parents reported poorer calculation skills for females with ADHD (Figure 4E). Males usually are outperforming females in most mathematic competencies (29). Thus, this finding appears related to gender/sex and not to the neurobiological mechanisms of ADHD.

### Gender/sex-sensitive implications for clinical practice

For family and developmental history, we found in our sample an overlap between females and males in the disorder-specific clinical presentation of parentally reported concerns, observations and experiences with their children with ADHD. Our findings support the recommendation that the disorder-specific therapeutical approach should be for females with ADHD similar to males with ADHD (30, 31), notably therapeutic decisions related to medication in females (32). We suggest that underlying neurobiological processes leading to ADHD lead to a stable presentation of a diagnosis-typical development.

Despite the overall impression of great overlap, we still found a couple of tendencies of gender/sex differences in our sample, especially differences in mathematical competencies and fine motor skills, also well described for neurotypically developing females (28, 29, 33). Other gender/sex differences like less support for female adolescents by adult caregivers or possible transgenerational female stereotypes may be considered by the clinician as well. We still suggest that therapeutic strategies to improve the individual achievement of children and adolescents with ADHD should be developed in a gender- and sex-sensitive manner.

### Limitations

Our study represents the number of matching male and female patients without comorbidity that could be recruited from about 10 years of treatment at a large child and adolescent psychiatry department. As our study excluded ADHD patients with comorbidities, which account for a large proportion of both males and females in clinical populations, statements for these patients are restricted. Other limitations of our study are non-blinded student interviewers. Further, applying correction for multiple comparisons would result in disappearing of most of the few differences found here. However, this does not contradict our main finding of strong overlap between boys and girls.

Taken together, a relatively small clinical study population may in first line generate a new hypothesis – in ADHD without comorbidity there are few differences in family and developmental history between boys and girls. Our study was an exploratory study and equipped with limited resources. However, our study is to our knowledge the first one to investigate gender/sex differences in family and developmental history in ADHD.

### Conclusion

Our study suggests that the majority of ADHD-specific signs and narratives reported by parents in the exploration of the family and developmental history are stable diagnostic markers regardless of gender or sex.

## Data availability statement

The raw data supporting the conclusions of this article will be made available by the authors, without undue reservation.

## Ethics statement

The studies involving human participants were reviewed and approved by the Ethics Committee of the University Hospital Carl Gustav Carus (reference number EK 295072016) and was performed in accordance with the ethical standards laid down in the 1964 Declaration of Helsinki and its later amendments. Participants and also their children gave written informed consent to participate. Written informed consent to participate in this study was provided by the participants’ legal guardian/next of kin.

## Author contributions

JW collected the data, interpreted the results, and wrote the paper. JZ collected the data and wrote the paper. VR wrote the paper. RW designed the study, supervised the collection of data, interpreted the results, and wrote the paper. All authors contributed to the article and approved the submitted version.

## Conflict of interest

The authors declare that the research was conducted in the absence of any commercial or financial relationships that could be construed as a potential conflict of interest.

## Publisher’s note

All claims expressed in this article are solely those of the authors and do not necessarily represent those of their affiliated organizations, or those of the publisher, the editors and the reviewers. Any product that may be evaluated in this article, or claim that may be made by its manufacturer, is not guaranteed or endorsed by the publisher.
