# Supplementary material for: Family and developmental history of female versus male adolescents with ADHD: diagnosis-specific overlap, few gender/sex differences
Source: Front Psychiatry. 2023 Jul 17;14:1072735. doi: 10.3389/fpsyt.2023.1072735 (PMC10390694; doi:10.3389/fpsyt.2023.1072735)

Recruitment and matching process

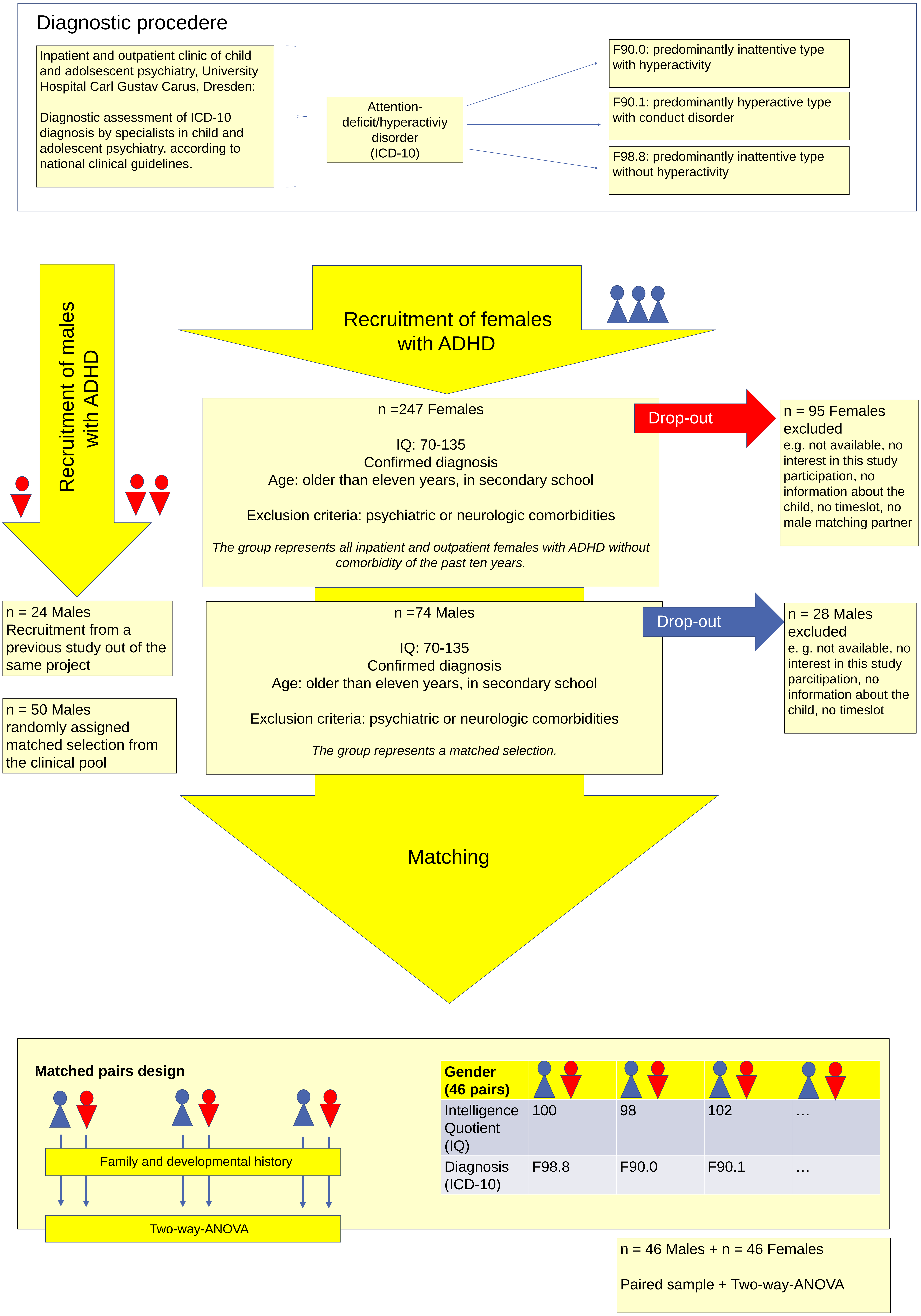

Supplement: Supplementary file 3 [file Data_Sheet_1.PDF]
